# LOFIT (Lifestyle front Office For Integrating lifestyle medicine in the Treatment of patients): a novel care model towards community-based options for lifestyle change—study protocol

**DOI:** 10.1186/s13063-022-06960-z

**Published:** 2023-02-17

**Authors:** Marlinde L. van Dijk, Leonie M. te Loo, Joyce Vrijsen, Inge van den Akker-Scheek, Sanne Westerveld, Marjan Annema, André van Beek, Jip van den Berg, Alexander L. Boerboom, Adrie Bouma, Martine de Bruijne, Jeroen Crasborn, Johanna M. van Dongen, Anouk Driessen, Karin Eijkelenkamp, Nies Goelema, Jasmijn Holla, Johan de Jong, Anoek de Joode, Arthur Kievit, Josine van’t Klooster, Hinke Kruizenga, Marike van der Leeden, Lilian Linders, Jenny Marks-Vieveen, Douwe Johannes Mulder, Femmy Muller, Femke van Nassau, Joske Nauta, Suzanne Oostvogels, Jessica Oude Sogtoen, Hidde P. van der Ploeg, Patrick Rijnbeek, Linda Schouten, Rhoda Schuling, Erik H. Serné, Simone Smuling, Maarten R. Soeters, Evert A. L. M. Verhagen, Johannes Zwerver, Rienk Dekker, Willem van Mechelen, Judith G. M. Jelsma

**Affiliations:** 1 Amsterdam UMC, VU University Medical Center, Department of Public and Occupational Health, Amsterdam Public Health research institute, Van der Boechorststraat 7, 1081BT Amsterdam, The Netherlands; 2 Department of Public and Occupational Health, Amsterdam UMC location Vrije Universiteit Amsterdam, De Boelelaan 1117, Amsterdam, The Netherlands; 3 Health Behaviors & Chronic Diseases, Amsterdam Public Health Research Institute, Amsterdam, The Netherlands; 4 Quality of Care, Amsterdam Public Health Research Institute, Amsterdam, The Netherlands; 5 Faculty of Health, Sports and Social Work, Inholland University of Applied Sciences, Haarlem, The Netherlands; 6 Department of Orthopedics, Groningen, University of Groningen, University Medical Center Groningen, Groningen, The Netherlands; 7 Department of Orthopedics, Ommelander Hospital Groningen, Scheemda, Groningen, The Netherlands; 8 Department of Endocrinology and Metabolic Diseases, University of Groningen, University Medical Center Groningen, Groningen, The Netherlands; 9 Department of Nephrology, University of Groningen, University Medical Center Groningen, Groningen, The Netherlands; 10 Department of Rehabilitation Medicine, University of Groningen, University Medical Center Groningen, Groningen, The Netherlands; 11 Health Insurance Expertise (formerly Zilveren Kruis), Utrecht, The Netherlands; 12 Department of Health Sciences, Faculty of Science, Vrije Universiteit Amsterdam, Amsterdam Public Health Research Institute, Amsterdam, The Netherlands; 13 Amsterdam Rehabilitation Research Centre, Reade, Amsterdam, The Netherlands; 14 Institute of Sports Studies, Hanze University of Applied Sciences, Groningen, The Netherlands; 15 Department of Orthopedics, Amsterdam UMC location University of Amsterdam, Meibergdreef 9, Amsterdam, The Netherlands; 16 Department of Strategy, Development and External Relations, University of Groningen, University Medical Center Groningen, Groningen, The Netherlands; 17 Department of Nutrition & Dietetics, Amsterdam UMC location Vrije Universiteit, De Boelelaan, 1117 Amsterdam, The Netherlands; 18 Department of Rehabilitation Medicine, Amsterdam Movement Sciences Research Institute, Amsterdam Public Health Research Institute, Amsterdam UMC location Vrije Universiteit, Amsterdam, The Netherlands; 19 Department of Anesthesiology, Amsterdam UMC location Vrije Universiteit, De Boelelaan, 1117 Amsterdam, The Netherlands; 20 Department of Internal Medicine, University of Groningen, University Medical Center Groningen, Groningen, The Netherlands; 21 Zilveren Kruis, Leiden, The Netherlands; 22 Menzis, Groningen, The Netherlands; 23 Huis voor de Sport in Groningen, Groningen, The Netherlands; 24 NL Actief, Ede, The Netherlands; 25 Team Sportservice Noord-Holland, Haarlem, The Netherlands; 26 Department of Internal Medicine, Amsterdam UMC location Vrije Universiteit, De Boelelaan, 1117 Amsterdam, The Netherlands; 27 Department of Internal Medicine, Amsterdam UMC location University of Amsterdam, Meibergdreef 9, Amsterdam, The Netherlands; 28 Center for Human Movement Sciences, University of Groningen, University Medical Center Groningen, Groningen, The Netherlands; 29 Sports Valley, Sports Medicine, Gelderse Vallei Hospital, Ede, The Netherlands

**Keywords:** Lifestyle, Lifestyle front office, Noncommunicable diseases, Delivery of health care, Health behaviour, Randomized controlled trial

## Abstract

**Background:**

A healthy lifestyle is indispensable for the prevention of noncommunicable diseases. However, lifestyle medicine is hampered by time constraints and competing priorities of treating physicians. A dedicated lifestyle front office (LFO) in secondary/tertiary care may provide an important contribution to optimize patient-centred lifestyle care and connect to lifestyle initiatives from the community. The LOFIT study aims to gain insight into the (cost-)effectiveness of the LFO.

**Methods:**

Two parallel pragmatic randomized controlled trials will be conducted for (cardio)vascular disorders (i.e. (at risk of) (cardio)vascular disease, diabetes) and musculoskeletal disorders (i.e. osteoarthritis, hip or knee prosthesis). Patients from three outpatient clinics in the Netherlands will be invited to participate in the study. Inclusion criteria are body mass index (BMI) ≥25 (kg/m^2^) and/or smoking. Participants will be randomly allocated to either the intervention group or a usual care control group. In total, we aim to include 552 patients, 276 in each trial divided over both treatment arms. Patients allocated to the intervention group will participate in a face-to-face motivational interviewing (MI) coaching session with a so-called lifestyle broker. The patient will be supported and guided towards suitable community-based lifestyle initiatives. A network communication platform will be used to communicate between the lifestyle broker, patient, referred community-based lifestyle initiative and/or other relevant stakeholders (e.g. general practitioner). The primary outcome measure is the adapted Fuster-BEWAT, a composite health risk and lifestyle score consisting of resting systolic and diastolic blood pressure, objectively measured physical activity and sitting time, BMI, fruit and vegetable consumption and smoking behaviour. Secondary outcomes include cardiometabolic markers, anthropometrics, health behaviours, psychological factors, patient-reported outcome measures (PROMs), cost-effectiveness measures and a mixed-method process evaluation. Data collection will be conducted at baseline, 3, 6, 9 and 12 months follow-up.

**Discussion:**

This study will gain insight into the (cost-)effectiveness of a novel care model in which patients under treatment in secondary or tertiary care are referred to community-based lifestyle initiatives to change their lifestyle.

**Trial registration:**

ISRCTN ISRCTN13046877**.** Registered 21 April 2022.

## Background

Lifestyle-related health risks are rising rampantly. Physical inactivity, sedentary behaviour, unhealthy diet, tobacco use and harmful use of alcohol are highly prevalent in modern society and are associated with overweight and obesity, increased blood pressure, increased serum cholesterol and ultimately the development of—avoidable—noncommunicable diseases (NCDs) [1, 2]. In the Netherlands, 89% of all deaths are attributed to NCDs, including cardiovascular disease (CVD), osteoarthritis and diabetes mellitus type 2 (DM2) [3]. It is no surprise that the Dutch annual direct health care costs are tremendous for people with NCDs; i.e. €11.6 billion for CVD, €1.4 billion for osteoarthritis and €1.6 billion for diabetes in 2019 [4]. It is expected that this situation will worsen in the coming decades, due to the ageing population and the steady increase of the worldwide obesity pandemic [5, 6]. It should be noted that the financial figures mentioned above do not include indirect costs, such as those due to productivity losses, and are thus a large underestimation of the actual societal cost of people with NCDs.

Guidelines advise healthcare professionals to encourage patients to eat healthier, be physically active regularly, stop smoking and limit alcohol use in the treatment of NCDs. Many interacting factors are associated with healthy behaviour, for instance, socioeconomic status including educational level [7]. One particularly important factor is the patient’s motivation [8]. Patients’ fear of disease progression and patients’ experiences of health complaints in daily life create a window of opportunity for the uptake of lifestyle changes by (re-)gaining some form of self-control over a healthy lifestyle [9]. However, health care professionals do not consistently use this opportunity to motivate the patient and explore in-depth options for lifestyle change. Reasons for this include competing priorities, lack of skills and knowledge and time constraints [10]. Another barrier is that doctors often do not feel confident in advising the patient and discussing lifestyle-related topics [11]. This fear of offending patients, disrupting patient-doctor relationships, and not knowing which patient is eligible for lifestyle counselling limit the total amount of lifestyle advice given by medical specialists to their patients [12]. Finally, healthcare professionals are often unaware of available lifestyle interventions and community-based initiatives (e.g. locally) to help patients change their lifestyle. This becomes more apparent in (academic) hospitals with a large adherence area. Consequently, integration of lifestyle medicine in daily clinical practice is hampered, and referral by healthcare professionals to hospital or community-based lifestyle initiatives is low, even though there are sufficient opportunities for health promotion activities in the community [13, 14].

To overcome these barriers to providing lifestyle advice lifestyle front office (LFO) in secondary/tertiary care might enhance integration of lifestyle medicine for patients living with NCDs. This LFO is a novel element in the existing care pathway that patients in clinical care follow. In a dedicated LFO, trained lifestyle brokers build motivation for lifestyle change in dialogue with the patient and refer patients to local community-based lifestyle change initiatives. An LFO likely improves the quality of clinical lifestyle care delivery because lifestyle brokers have dedicated time and are both skilled and qualified to deliver lifestyle counselling. Furthermore, the visibility of an LFO is expected to create an in-hospital sense of importance for a healthy lifestyle for both professionals and patients.

This study aims to evaluate the effectiveness and cost-effectiveness of an LFO in routine hospital care. It is hypothesized that the LFO model of care will increase the uptake of a healthy lifestyle, consequently reduce disease symptoms, medical complications and the amount of prescribed medication and prevent the development of (other) NCDs and thus lower healthcare and societal costs in comparison to usual care [15].

## Methods

### Study design

**Fig. 1:**
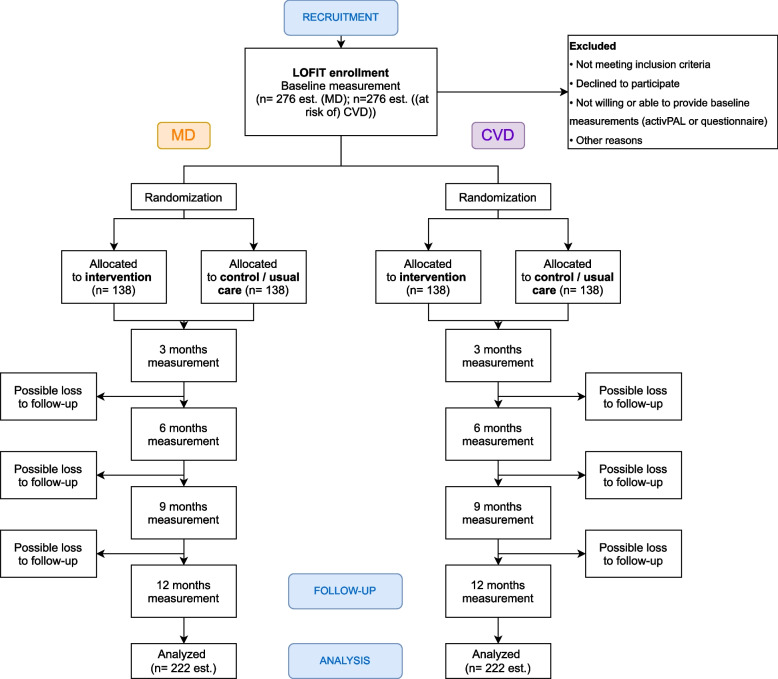
Trial schedule. Abbreviations: MD: musculoskeletal disorders; CVD: (cardio) vascular disorders

This multicentre study consists of two separate, parallel conducted, pragmatic randomized controlled trials (RCT). One RCT for patients who live with or have a high risk on (cardio) vascular disease and one RCT for patients who live with osteoarthritis or with a total hip or knee prosthesis. Patients will be recruited from two departments (Internal Medicine and Orthopedics) in two large university medical centres (Amsterdam UMC, UMC Groningen) and from one smaller rural hospital in The Netherlands (Ommelander Ziekenhuis Groningen). Measurements are taken at baseline and at 3, 6, 9 and 12 months follow-up. In total, we aim to include 552 patients across both RCTs. Figure 1 shows the trial schedule according to the CONSORT template. The study was approved by the respective ethical committees before the start (VUmc Amsterdam: 2021.0712; UMC Groningen: 2022.182; OZG: 331819).

### Participants

Patients with (an increased risk for) cardiovascular disorders (i.e. cardiovascular disease, hypertension, high cholesterol, diabetes mellitus I and II) or with musculoskeletal disorders (i.e. osteoarthritis, total knee or hip prosthesis) aged ≥18 years are eligible for inclusion. Further inclusion criteria are (1) having a body mass index (BMI) of ≥ 25 kg/m^2^ and/or (2) smoking. Patients will be excluded if they are not able to walk at least 100 m safely (e.g. wheelchair-bound), are pregnant, are cognitively unable to comply with a healthy lifestyle intervention referral or to complete study measurements or are not able to communicate in the Dutch or English language.

#### Sample size and power calculation

The primary outcome of this study is a composite health risk and lifestyle score (i.e. adapted Fuster-BEWAT score). The numbers needed in each trial arm (80% power, 5% significance, two-tailed alpha) were calculated assuming a 20% drop-out. To detect a difference of 1.45 change in the adapted Fuster-BEWAT score (with a standard deviation of 3.79), 138 patients will be allocated to each arm (thus a total of 276 patients per subtrial) [16, 17]. The two randomized controlled trials (musculoskeletal and cardiovascular) will be conducted in parallel. Therefore, the LOFIT trial must include a total of 552 patients.

### Recruitment and screening

Ongoing recruitment is planned between April 2022 and March 2023. Health care professionals in the hospital will be informed about the LFO and the study during regular meetings, flyers, pocket cards with information about the LFO and a website. Each participating department has a spokesperson (i.e. a local champion) who is tasked with bringing the LFO to the attention of his or her colleagues. Attention for the LFO is also created by the lifestyle broker during multiple disciplinary consultation meetings. Patients will be primarily informed about the study purpose by their health care professional, but also through flyers, narrowcasting in the waiting room and a website (www.lofitleefstijlloket.nl). Health care professionals can refer patients through the electronic patient file system. Subsequently, patients will receive the study information brochure and have the opportunity to take study participation into consideration and to ask questions about the study to a study staff member.

Patients who are interested in participating will be scheduled for a baseline measurement appointment. At baseline, all participants will be asked to sign an informed consent form by the researchers in which they consent to participate in the study, including the collection of data during study measurements, facultative blood sampling and facultative extraction of relevant data from medical records. Participants may additionally consent to an audio recording of the session with the lifestyle broker (in case of randomization to the intervention group). In- and exclusion criteria will be checked during the baseline measurement. Study participation is voluntary, and participants can withdraw from the study at any time without giving any reason and without affecting usual care. Patients’ privacy and data are protected, and all data will be processed after pseudonymization, adhering to standard procedures of the participating medical centres. A screening log will contain all patients screened for the study and the reason why they were excluded from randomization or why they were unwilling to participate, if applicable, to allow the consort diagram to be completed.

### Randomization and blinding

Randomization occurs separately for both trials, and patients will be randomly allocated to receive the LOFIT intervention or usual care in a 1:1 ratio. A computerized random number generator (Sealed Envelope) draws up an allocation schedule pre-stratified for hospital centres using randomized permuted blocks of sizes 4 and 6 [18]. Sealed opaque envelopes that contain the group to which a patient is allocated will be prepared. The outcome of the allocation will be reported by research staff to the participant and healthcare professional before the start of the intervention to keep those involved with measurements blinded. A design in which individuals will be randomized was chosen instead of a cluster-randomized design, as minimal contamination between the intervention and usual care group is expected. Due to the nature of the intervention, participants cannot be blinded for the treatment allocation but are asked not to reveal information about their intervention allocation to the measurement team. Field work staff who perform the follow-up assessments will be kept blinded to group allocation. Researchers who process and clean quantitative data will be blinded for group allocation.

### Lifestyle intervention

#### Intervention

After randomization to the intervention arm, an individual session of approximately 45 min with the lifestyle broker will be scheduled. Patients can visit the LFO in the hospital or schedule an appointment for a telephone or video call. The goal of this session is to establish patient’s motivation for lifestyle change and consider the capabilities and opportunities of the patient, in order to refer the patient to a suitable community-based lifestyle initiative which will facilitate and maintain behavioural change.

The lifestyle broker will guide the patient in a dialogue, while following six steps based on Motivational Interviewing (MI) [19] and the COM-B model of behaviour [20]. In these six steps, the patient and the lifestyle broker will (1) engage with each other. The basic attitude of the lifestyle broker towards the patient is sincere, emphatic, respectful and non-judging. On this condition, patients are much more likely to talk freely about their struggles with lifestyle issues and will be more prepared to find solutions together [21]; (2) determine the preferred behaviour to change (i.e. physical activity, healthy eating, smoking, stress, alcohol, sleep); (3) discuss (a) pros and cons regarding the current lifestyle and regarding the intended lifestyle change; (b) the capabilities and opportunities in the context of the patient (i.e. level of (health) literacy, language barriers, social support and/or social barriers, psychological barriers, cultural and religious factors, personal financial situation); (c) the patient’s preferences regarding lifestyle change supervision (i.e. group, individual, none); (4) discuss which neighbourhood and community-based initiative is most suitable for the patient, taking into account all factors from step 3; (5) make an specific action plan to which the patients commits; (6) operationalize the action plan, which leads to a referral to a community-based lifestyle initiative (e.g. combined lifestyle intervention programs, stop-smoking coaches, lifestyle coaches, physical activity coaches, psychologists, dieticians, physiotherapist, general practitioners, local walking groups, (prevention) fitness centre, social domain, debt counselling, addiction treatment) and to practical arrangements about follow-up.

**Fig. 2:**
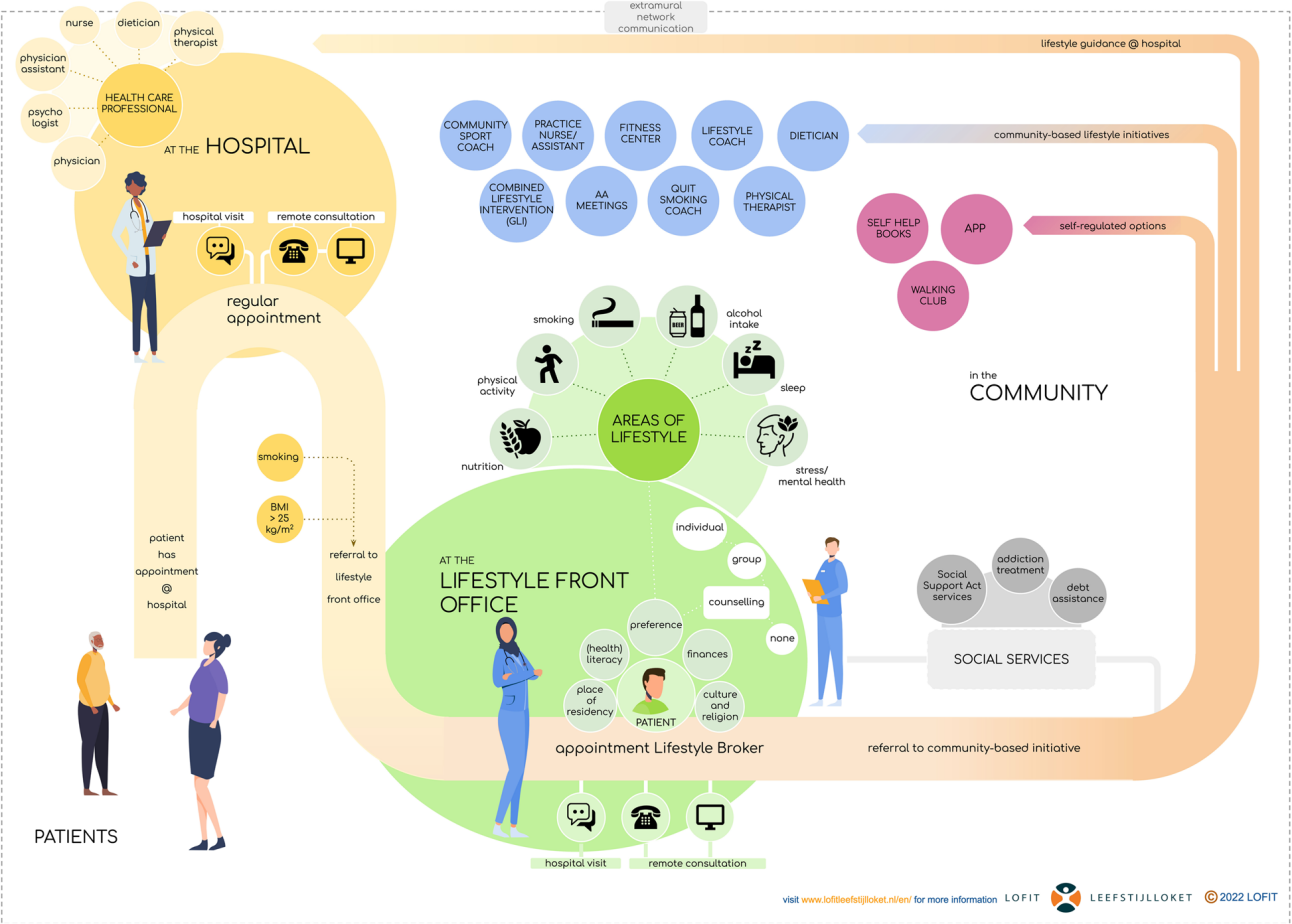
Overview of the patient journey

After referral to a community-based lifestyle initiative, the lifestyle broker will monitor progress and will maintain contact through an online secured network communication platform (i.e. cBoards, Caresharing BV [Ltd.], The Netherlands) as long as the patient is under treatment in the hospital. This platform enables the communication between the patient, community-based lifestyle initiatives and other relevant stakeholders (e.g. general practitioner, informal caregiver). The frequency of contact between the involved parties is tailored to patients’ preferences, needs and scheduled hospital appointments. The duration of the intervention depends on the community-based lifestyle initiative referred to. The lifestyle broker will inform the healthcare professional in the hospital regularly about the progress of their patients via the electronic patient file system. Figure 2 shows the patient journey that patients will undertake when randomized in the intervention group.

#### Lifestyle broker

The lifestyle broker is a professional with at least a polytechnic tertiary education degree. Furthermore, the lifestyle broker is familiar with lifestyle counselling and medical terminology, has basic knowledge of medical diseases, has experience with patient care and is aware of available community-based lifestyle initiatives. For this study, the lifestyle brokers will be further trained in MI during a 3-day MI training by a certified MI trainer; will receive regular feedback on at least five audio-recorded conversations with patients to obtain an adequate proficiency level; will have regular peer intervision meetings to facilitate peer-learning experiences with other lifestyle brokers; and will participate in an online community platform, in which support tools are integrated, and experiences can be exchanged between lifestyle brokers [22, 23].

### Control group

Control group patients will receive care as usual from their healthcare professional(s). In practice, this entails that provision of lifestyle advice is highly dependent on individual healthcare professionals. It is impossible to prevent patients in the control group from organizing guidance towards a healthy lifestyle by themselves. At 12 months follow-up, the control group will be asked by questionnaire if they have participated in some form of health promotion activity over the past 12 months, to explain possible lifestyle change in the control group.

### Data collection

**Fig. 3:**
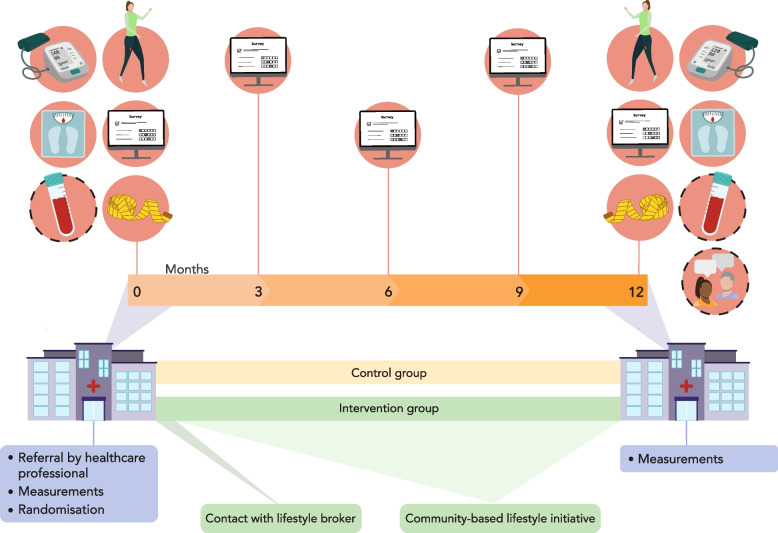
SPIRIT figure for the schedule of enrolment, interventions and assessments. See Table 1 for a more detailed overview of assessments incl. timepoints

**Fig. 4:**
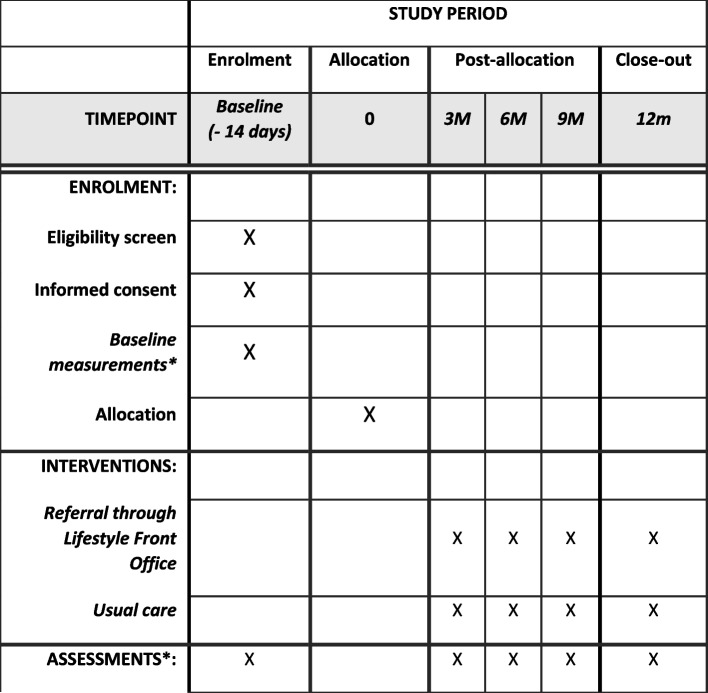
Visualization of data collection over 12 months

Data will be collected from patients at five time points: at baseline and at 3 months (only costs, health-related quality of life and process evaluation measures; online questionnaire), 6 months, 9 months (both only costs and health-related quality of life; online questionnaire) and 12 months follow-up. Table 1 gives a detailed overview of the measurements. Patients are expected at the hospital for measurements at baseline and 12 months follow-up. Figure 3 shows the SPIRIT figure that visualizes all moments of data collection. Visits are scheduled as much as possible in conjunction with usual care visits to manage the time of participating patients efficiently. Anthropometric measurements will be performed, and blood will be taken. Participants will be asked to wear a small, lightweight, inertial measurement unit (IMU) (i.e. activPAL™) for 9 days consecutively to objectively measure steps and sitting time. Patients receive a text on the ninth day with a reminder to remove the IMU. After 9 days, patients will be asked to complete a questionnaire per email, redirecting participants to a web-based questionnaire format (Castor EDC, Amsterdam, The Netherlands). Automatic reminders are sent to participants if the online questionnaire was not filled in within 1 week. In order to minimize the amount of missing data, validation rules were implemented in the online questionnaire. Data from the medical records will be retrieved at 12 monthly follow-ups. Figure 4 is a visualization of types of data collection over 12 months. Data monitoring and audit will follow Amsterdam UMC (sponsor) local standards (i.e. data monitoring after 10% of anticipated inclusion rate with a yearly follow-up by independent observant).

**Table 1:** Samples and measurements

	Method/sample used	Baseline	3M	6M	9M	12M
PRIMARY OUTCOME						
Health risk and lifestyle (Fuster-BEWAT) [24]	Adapted Fuster-BEWAT	x				x
SECONDARY OUTCOMES						
CARDIOMETABOLIC MARKERS						
Fasting plasma glucose, lipids (triglycerides, total cholesterol, HDL cholesterol, LDL cholesterol), insulin, HbA1C, liver function (GGT, ALT, AST), kidney function (creatinine)	Blood sample	x				x
ANTHROPOMETRIC						
Body height	Stadiometer (once)	x				x
Body weight	Scale (once)	x				x
Waist circumference	Tape (twice)	x				x
Neck circumference	Tape (once)	x				x
Resting blood pressure (systolic, diastolic)	Once	x				x
BEHAVIOUR						
*Objectively measured lifestyle behaviour*						
Sitting time, upright time and step count	activPAL	x				x
*Self-reported lifestyle behaviour*						
Dietary intake and quality	Questionnaire	x				x
Alcohol intake (AUDIT) [25, 26]	Questionnaire	x				x
Sedentary behaviour (Marshall) [27]	Questionnaire	x				x
Physical activity (IPAQ-SF) [28–30]	Questionnaire	x				x
Fitness (FitMax) [31]	Questionnaire	x				x
Sleep insomnia (ISI) [32–34]	Questionnaire	x				x
Sleep quality (Brief-PSQI) [35]	Questionnaire	x				x
Obstructive Sleep Apnea Syndrome (OSAS) [36, 37]	Questionnaire	x				x
Smoking status (FTND) [38]	Questionnaire	x				x
PSYCHOLOGICAL						
Wellbeing (Cantril ladder) [39]	Questionnaire	x				x
Health-related Quality of Life (EQ-5D-5L) [40, 41]	Questionnaire	x	x	x	x	x
Resilience (BRS) [42]	Questionnaire	x				x
General self-efficacy scale (GSES) [43, 44]	Questionnaire	x				x
Stage of change [45]	Questionnaire	x				x
PROMs						
Functional limitations (HOOS-PS /KOOS-PS) [46]^1^	Questionnaire	x				x
SOCIAL DEMOGRAPHICS						
Age	Questionnaire	x				
Gender	Questionnaire	x				
Ethnicity	Questionnaire	x				
Marital status	Questionnaire	x				
Number of children	Questionnaire	x				
Education	Questionnaire	x				
Household income	Questionnaire	x				
Employment status	Questionnaire	x				
Postal code	Questionnaire	x				
Health literacy (SBSQ) [47]	Questionnaire	x				
ASA physical status classification [48]	Electronic Patient File	x				
Family history of diseases	Questionnaire	x				
Comorbidity (CCI) [49]	Electronic Patient File	x				
COST-EFFECTIVENESS						
Productivity and healthcare use (iPCQ, iMCQ) [40, 50]	Questionnaire	x	x	x	x	x
Consequences for employment	Questionnaire	x	x	x	x	x
Medication use	Questionnaire	x	x	x	x	x
Travel costs to hospital	Questionnaire	x				
PROCESS EVALUATION						
Implementation, context, mechanism of impact [51]	Questionnaire, interviews, data registration	x	x			x

^1^Only used by patients who have osteoarthritis

### Primary outcome

The adapted Fuster-BEWAT score is a composite health risk and lifestyle score, which consists of six components: resting blood pressure (mmHg), objectively measured physical activity (steps/24h), objectively measured sitting time (time/24h), body mass index (BMI), fruit and vegetable consumption (servings/24h) and smoking (units/24h). For each component, the patient will receive a risk score (0–4 points), where the highest score reflects public health guidelines. The total adapted Fuster-BEWAT score ranges from 0 to 24, with a higher score indicating lower risk. The original Fuster-BEWAT has been validated to predict the presence and extent of subclinical atherosclerosis, which was measured in a prospective cohort study of 4184 asymptomatic Spanish bank employees [24]. The adapted Fuster-BEWAT has recently been tested in a lifestyle intervention on responsiveness and effects of a lifestyle intervention in a Spanish bank population [16]. The adapted Fuster-BEWAT does not require obtaining blood samples, making this measure a highly feasible outcome and progress marker for an LFO (Table 2).

**Table 2:** Adapted Fuster-BEWAT composite health risk and lifestyle score

Score	0	1	2	3	4
Systolic/diastolic blood pressure^a^ (mmHg)	≥140/90	134–139/ 87–89	128–133/ 84–86	121–127/ 81–83	≤120/80
Physical activity (steps/24h)	<5500	5500–6999	7000–8499	8500–9999	≥10,000
Sitting (h/24h)	≥12.5	11 – < 12.5	9.5 – < 11	8 – < 9.5	<8
Body mass index^b^ (kg/m^2^)	≥32	30–31.9	27–29.9	25–26.9	<25
Fruit and vegetable consumption (servings/24h)	≤1	2	3	4	≥5
Smoking (units/24h)	>20	10–20	1–9	<1	0

^a^If systolic and diastolic blood pressure does not fall in the same category, then the participant is assigned to the category with the relatively highest blood pressure (i.e. systolic or diastolic)

^b^At follow-up visits, a 5% decrease in BMI will add 1 extra point to the BMI score, except for those participants who, due to this decrease, already have changed BMI categories since baseline or who are already in the normal bodyweight category (BMI<25kg/m^2^). Similarly, a 5% increase in BMI at follow-up will result in 1 point less in the BMI score, except for participants who have changed BMI categories since baseline or participants with a BMI≥ 32 kg/m^2^

### Secondary outcomes

#### Cardiometabolic biomarkers

Blood samples will be taken at baseline and at 12 months follow-up. If patients opt-out of blood collection, blood samples collected in the previous month for usual care purposes will be recorded if available. Patients will be asked to fast for at least 8 h prior to blood collection. The samples will be centrifuged, and the following markers will be analysed: fasting plasma glucose, serum fasting insulin, hemoglobin A1C (HbA1C), serum lipids (triglycerides, total cholesterol, high-density lipoprotein cholesterol (HDL) and low-density lipoprotein cholesterol (LDL)), serum liver function enzymes (gamma-glutamyl transferase (GGT), alanine aminotransferase (ALT), aspartate aminotransferase (AST)), and kidney function (serum creatinine and estimated glomerular filtration rate).

Biomarkers will be used to calculate a bio-medical cardiometabolic risk score, using variables with strong associations to incident cardiovascular and metabolic disease [52]. The cardiometabolic risk score will be calculated as follows: (mean of *z*-scores for fasting glucose, fasting insulin, HbA1c, total cholesterol, (−)HDL cholesterol, triglyceride, ALT, AST, GGT, systolic blood pressure and diastolic blood pressure) × 10. If laboratory values deviate from hospital reference ranges or the Dutch College of General Practitioners’ (NHG) practice guidelines [53–56], the referring healthcare worker (department of internal medicine) or general practitioner (department of orthopedics) will be informed for further action. This will be documented as an adverse event (AE).

#### Anthropometric measures

The following anthropometric measures will be carried out. Body height will be measured to the nearest centimetre at baseline only with a stadiometer (SECA 206, SECA, Birmingham, UK). Body weight to the nearest 0.1kg without shoes and light clothes will be measured using calibrated electronic scales (SECA 877). Body height and weight will be used to calculate BMI (kg/m^2^). BMI (i.e. categorized into >31.9 (0); 30–31.9 (1); 27–29.9 (2); 25–26.9 (3); <25 (4)) will be used for the adapted Fuster-BEWAT score. Waist circumference will be measured twice to the nearest 0.1 cm with a SECA 201 measure (no shirt on), midway between the lowest ribcage and the iliac crest. If the difference between the two measures is >0.5 cm, a 3rd measurement will be conducted. The mean will be calculated from the two nearest measurements. Body height and waist circumference will be used to calculate the waist-to-height ratio [57]. Neck circumference will be measured once to the nearest 0.1cm with a SECA 201 measure in a standing relaxed upright position between the mid-cervical spine and mid-anterior neck [58]. For resting blood pressure, Omron M6 blood pressure monitor was used after 5 min of sitting still. If measured systolic blood pressure is >139 mmHg and/or measured diastolic blood pressure is >89 mmHg, two additional measurements will be taken and recorded. A mean will be calculated from the second and third measurements. Both systolic and diastolic blood pressure (i.e. categorized into systolic/diastolic: >140/90 (0); 134–139/87–89 (1); 128–133/84–86 (2); 121–127/81–83 (3); <121/81 (4)) will be used for the adapted Fuster-BEWAT score. All equipment used in the different hospitals is identical.

#### Health behaviour

Objectively measured sitting time, upright time, and the number of steps will be measured using the activPAL™ micro (PAL Technologies Ltd., Glasgow, UK) physical activity monitor, worn on the right front thigh for seven consecutive full days. This is a small device weighing 9 g, made waterproof with a nitrile sleeve. It will be secured with adhesive hypoallergenic thin plastic film (Tegaderm Roll, 3M). Participants will receive verbal instructions and will be shown how to wear the activPAL™ device and to correctly (re)attach it (e.g. after swimming and bathing). The activPAL™ has good measurement properties in adults for assessing sitting time, standing time, stepping time and the number of steps/day, and should be worn for at least 4 days [59]. Variables of interest calculated from the activPAL™ data include sitting time, standing time, stepping time, the number of bouts and time of prolonged sitting (>30 min; >60 min; >120 min), steps and the number of sit-to-stand transitions. All these outcomes will be calculated and averaged to comprise 24-h physical activity results. Steps per 24h (i.e. categorized into: <5500 (0); 5500–6999 (1); 7000–8499 (2); 8500–9999 (3); >10,000 (4)) and sitting time (i.e. categorized into daily: ≥12.5 (0); 11–<12.5 (1); 9.5–<11 (2); 8–<9.5 (3); <8 (4)) will be used for the adapted Fuster-BEWAT score.

Self-reported cardiorespiratory fitness will be assessed with the FitMax [31]. This is a questionnaire consisting of three single-answer questions about the maximum capacity of walking, climbing stairs and cycling. The questionnaire has a scale range from 0 to 13 to rate maximum capacity of walking, a scale range from 0 to 11 for the maximum capacity of cycling and a scale range from 0 to 10 to rate the maximum capacity of stair climbing. The FitMax is strongly correlated (*r*=0.95) with peak oxygen uptake as measured with cardiopulmonary exercise testing in healthy people as well as patients [31].

Self-reported physical activity will be assessed with the International Physical Activity Questionnaire – Short Form (IPAQ-SF), measuring walking, moderate and vigorous-intensity physical activity, and with good validity across many populations, including osteoarthritis [28–30].

Self-reported sedentary time will be assessed with the Marshall questionnaire with 10 items, which has shown good reliability and validity for context-specific sitting when studied against accelerometry [27]. Total and domain-specific sitting time in minutes will be assessed (i.e. sitting during transport, at work, while watching television, while using the computer for leisure, and during other leisure activities) during a week and weekend day.

Self-reported dietary data will be measured with 18 items selected from the Dutch Food Frequency questionnaire (FFQ) [60]. Frequency of daily, weekly or monthly intake of the following foods and drinks will be obtained: fruit and vegetables, legumes, unsalted nuts, fish, red and processed meat, sugary drinks and sweets/cookies/chips/cake/chocolate. Furthermore, the dietary quality of the type of consumed bread, pasta, rice (i.e. wholegrain or white) and type of butter and preparation fat will be categorized. We will assess the frequency of meals and special diets. All these variables will be combined into one overall dietary quality score based on previous quality index score cards and the Dutch dietary guideline [61–63]. Table 3 provides threshold scores for 9 areas corresponding to the Dutch dietary guidelines and one additional item for unhealthy snacks. The total score ranges from 0 to 10, where a higher score indicates better adherence to dietary guidelines. Fruit and vegetable consumption (i.e. categorized into daily threshold points: 0 or 1 (0); 2 (1); 3 (2); 4 (3); 5 or more (4)) will be used for the adapted Fuster-BEWAT score.

**Table 3:** Components and Dutch Healthy Diet index 2015 (DHD15 index) and threshold score

	Component	Dutch dietary guidelines 2015^(61, 63)^	Threshold (1 point)
1	Vegetables	Eat at least 200 g of vegetables daily	≥200 g daily
2	Fruit	Eat at least 200 g of fruit daily	≥2 pieces daily
3	Wholegrain products	Replace refined cereal products with whole grain products	No consumption of refined products
4	Legumes	Eat legumes weekly	≥1 week
5	Nuts	Eat at least 15 g of unsalted nuts daily	≥105 g week
6	Fish	Eat one serving of fish weekly, preferably oily fish	≥1 week
7	Fats and oils	Replace butter, hard margarines and cooking fats with soft margarines, liquid cooking fats and vegetable oils	No consumption of butter, hard margarines and cooking fats
8	Red and processed meat	Limit consumption of red and processed meat	≤2 days of red meat a week; and <1 day of processed meat a week
9	Sweetened beverages	Limit consumption of sweetened beverages	<1 glass a day
Additional to the Dutch dietary guideline			
10	Unhealthy snacks	Limit intake of sweets, chips, pretzels, cookies, gingerbread, cake, pie, chocolate(s)	<3 times a week

Alcohol intake will be assessed with the Alcohol Use Disorders Identification Test (AUDIT) [25, 26]. This is a 10-item questionnaire that explores alcohol consumption, potential dependency and the experience of alcohol-related harm. Items are scored on a 5-point scale, in which a higher score correlates with more harmful alcohol consumption and a higher likelihood of dependence.

Sleep will be assessed with three instruments. For sleep quality, the Brief Pittsburgh Sleep Quality Index (B-PSQI) will be used. This is a reliable instrument measuring sleep duration, efficiency, latency, and disturbances using 6 questions [35]. Sleep efficiency will be calculated, and 5 questions are scored, in which the total range is 0–15. A higher score indicates worse sleep quality. Sleep insomnia will be assessed with the Insomnia Severity Index (ISI) to quantify the severity of insomnia [32–34]. Items are scored on a 5-point scale, with a total score ranging from 0 to 28. A higher score indicates more severe insomnia. The instrument is deemed valid to assess change over time. For assessment of obstructive sleep apnea (OSA), the STOP-BANG Sleep Apnea questionnaire will be used [36, 37]. This questionnaire provides a risk score for OSA based on questions that relate to the clinical features of sleep apnea in combination with age, gender, BMI and neck circumference. Items are scored yes or no, and the total score ranges from 0 to 8, in which a higher score indicates a higher risk of OSA.

Smoking behaviour will be assessed, including the date of quitting and the amount of current consumption. Number of cigarettes smoked per day (i.e. >20 (0); 10–20 (1); 1–9 (2); <1 (3); 0 (4)) will be used for the adapted Fuster-BEWAT score. Current smokers will be asked to fill out the six-item Fagerström Test for Nicotine Dependence (FTND). This test explores physical addiction to nicotine [38].

#### Psychological measures

Wellbeing will be measured using the Cantril ladder, a visual scale on which participants have to score their life [39]. The score ranges from a 0 for “worst possible life” to a 10 for “best possible life”. The General Self-Efficacy scale (GSE) will be used to measure self-efficacy [43, 44]. This is a 10-item questionnaire measuring a general sense of perceived self-efficacy to predict coping with daily hassle and adaptation after experiencing all kinds of stressful life events on a 4-point Likert scale (1=Not at all true; 4= Exactly true). Resilience will be assessed with the Brief Resilience Scale (BRS) [42]. This is a 6-item questionnaire measuring the degree of individual resilience on a 5-point Likert scale (1=strongly disagree; 5=strongly agree). A stages of change questionnaire will assess in what stage of behavioural change the participant is based on the transtheoretical model [45]. For smoking cessation, alcohol consumption, nutritional intake, physical activity, sedentary behaviour and sleep behaviour, one item was constructed with 6 response options, each reflecting one of the following stages: pre-contemplation (e.g. “I am physically active for less than 150 minutes per week and have not thought about changing”), contemplation (e.g. “I drink 7 or more alcoholic drinks a week, but I’ve thought about reducing my consumption”), preparation (e.g. “I want to quit smoking and I have begun to reduce the number of cigarettes that I smoke”), action (e.g. “I sit for less than 8 hours a day, but have been doing so for less than 6 months”), maintenance (e.g. “I eat according to the Dutch nutritional guideline and am doing so for more than 6 months”) or termination (e.g. “I am physically active for more than 150 minutes per week and I am doing so for many years. It is part of my lifestyle”). Health-related quality of life will be measured using the EQ-5D-5L. The EQ-5D-5L consists of five health dimensions; mobility, self-care, anxiety/depression, pain/discomfort and daily activities. Patients will be asked to rate their health-related quality of life on a 5-point scale as; no, slight, moderate, severe problems and unable to perform [40]. The patients’ EQ-5D-5L health states will be converted into utility scores, ranging from 0 (“death”) to 1 (“optimal health”), using the Dutch 5L value set [41]. For the economic evaluation, quality-adjusted life years (QALYs) will be estimated by multiplying the patients’ time spent in a certain state by the respective utility value.

#### Patient-reported outcome measures (PROMs)

For osteoarthritis, the Knee injury and Osteoarthritis Outcome Score Physical Function Short Form (KOOS-PS) and the Hip disability and Osteoarthritis Outcome Score Physical Function Short Form (HOOS-PS) have been developed from the original long version intended to evaluate the functional status of patients with knee/hip osteoarthritis. The HOOS-PS is a 5-item questionnaire, and the KOOS-PS is a 7-item questionnaire. Standardized response options are given, and each question is scored from 0 to 4 (on a 5-point Likert scale). Consequently, a normalized score, ranging from 0 to 100, is calculated (0 indicating extreme symptoms and 100 indicating no symptoms). The long Dutch version has been proven to be valid and reliable [46]. The HOOS-PSF and KOOS-PS are globally used as outcome measurement for a variety of studies and registries [64, 65].

#### Sociodemographic

At baseline, all participants will be asked to complete questions regarding demographic characteristics (i.e. age, gender, ethnicity, education, marital status, current employment status, income, number of children) and about a family history of diseases. Data on comorbidity will be retrieved from electronic patient files. For the classification of comorbidity, the Charlson Comorbidity Index (CCI) will be used [49]. Health literacy will be assessed with a 3-item Set of Brief Screening Questions (SBSQ) measured on a 5-point Likert scale (1=extremely; 5=not at all) to indicate low, marginal and high health literacy [47]). American Society of Anaesthesiologists (ASA) physical status classification will be based on the recording in the electronic patient file and is categorized as healthy (1); mild systemic disease (2); severe systemic disease (3); a severe systemic disease that is a constant threat to life (4); a moribund person not expected to survive without operation (5) [48].

#### Cost-effectiveness

This study will include a cost-effectiveness evaluation of an LFO in comparison to usual care regarding the primary outcome (i.e. adapted Fuster-BEWAT) and QALYs. Data on resource utilization will be collected using self-report questionnaires based on the iMTA productivity Cost and Medical Consumption questionnaires (iPCQ and iMCQ) [40, 50]. These questionnaires will assess utilization of healthcare services (i.e. number of visits to general practitioner, allied health professionals or complementary healthcare providers; number of ambulatory visits at a hospital or other health care organizations; admission to a hospital or other health care organization), medication use, the use of informal care, unpaid productivity losses as well as absenteeism (i.e. sick leave) and presenteeism (i.e. reduced productivity while being ill at work). Costs for the delivery of LOFIT will be estimated, applying a bottom-up micro-costing approach using—amongst others—costs reported by the hospital (i.e. recruitment of participants, implementation and delivery of the program) and the research group (i.e. preparation and start-up, and materials) [66]. The use of other healthcare services will be valued using Dutch standard costs and prices derived from www.medicijnkosten.nl. Informal care (i.e. care by family and friends) and unpaid productivity losses (i.e. costs associated with reduced productivity levels related to unpaid activities, such as volunteer work) will be valued using a recommended Dutch shadow price. Absenteeism will be valued according to the Friction Cost Approach and using gender-specific price weights. Presenteeism will be valued using gender-specific price weights.

#### Process evaluation

An extensive process evaluation will be conducted to investigate implementation processes during the two trials, to prepare for sustainable implementation of the LFO beyond the research setting. We will investigate the pathways along which the intervention affects outcomes, and facilitators and barriers for adoption, implementation and continuation. This process evaluation will be a mixed-method design, will be based on the UK MRC guidance on process evaluation of complex interventions [51] and will include data on context, implementation and mechanisms of impact. Data will be gathered from participants, lifestyle brokers, healthcare professionals and community-based lifestyle initiatives. Researchers will keep field notes of relevant information from phone calls, email and measurement observations. Table 4 presents an overview of the process evaluation objectives and methods used, ranked according to the domain context, implementation and mechanism of impact.

**Table 4:** Overview of the process evaluation objectives, methods used and timing ordered according the three domains

Research objectives		Quantitative methods							Qualitative methods						
		Q participant Baseline	Q participants 3M	Q participants 12M	Q health care professional 6M	Q community-based lifestyle initiative 12M	Q LsB - post training	Audiorecording session LsB	Electronic health records (0–12M)	IINT participant - post intervention (*n*=20)	INT LsB 12M (*n*=all)	INT health care professional 6M (*n*=10)	INT Community-based initiatives 12M (*n*=10)	INT broad context (ongoing)	Field notes researchers
DOMAIN 1: CONTEXT OF THE LIFESTYLE FRONT OFFICE															
Characteristics															
1.1	Characteristics of Lifestyle Broker (e.g. background, demographic characteristics, skills and experience)						X								X
1.2	Characteristics of community-based lifestyle initiatives (e.g. details initiative, size, facilities)								X						X
Facilitating factors and barriers to delivery of LOFIT															
1.3	Perceived barriers and facilitators to implementation of the LOFIT intervention*									X	X	X	X	X	
DOMAIN 2: IMPLEMENTATION OF THE LOFIT APPROACH															
Recruitment															
2.1	Sources and procedures for recruitment of health care professionals in participating departments														X
2.2	Sources and procedures for recruitment of Lifestyle Broker for LFO										X				X
2.3	Sources and procedures for recruitment of patients	X								X					X
2.4	Patients’ reported reasons for (not) joining, continuing with or withdrawal from the LOFIT intervention^1^	X								X					X
2.5	Health care professionals’ reported reasons for (non) participation, continuing with or withdrawal from the LOFIT intervention^1^				X							X			
Delivery															
2.6	[dose] The amount of delivered LOFIT intervention^1^		X	X					X	X		X	X		
2.7	[fidelity] The extent to which the LOFIT intervention^1^ was delivered as intended.		X	X	X	X		X	X		X	X	X		X
2.8	[dose] Stakeholder participation in the implementation strategies (e.g. amount of attended instruction meetings)				X	X									X
2.9	[fidelity] The extent to which the implementation strategies were used as intended by stakeholders (e.g. using pocket card and flyers as intended)				X	X					X	X	X		X
Reach															
2.10	Characteristics of patients (e.g. demographics and health risk profile).	X													
DOMAIN 3: MECHANISMS OF IMPACT															
Experiences with the LOFIT program and usefulness of elements															
3.1	Patients’ views and experiences of the LOFIT intervention^1^.		X	X						X					
3.2	Lifestyle brokers’ views and experiences of the LOFIT intervention^1^.										X				
3.3	Health care professionals’ views and experiences of the LOFIT intervention^1^.				X							X			
3.4	Community-based lifestyle initiatives’ views and experiences of the LOFIT intervention^1^.					X							X		

Abbreviations: *LFO* lifestyle front office, *LsB* lifestyle broker, *INT* interview, *Q* questionnaire, *EPD* Electronic Patient Record

^1^LOFIT intervention program is defined as follows: • A—Scheduling appointment in hospital; • B—Referral of patients to LFO: recruiting/referring patients for LFO; • C—LFO appointment: recruitment and training of lifestyle brokers, organization and delivery of sessions within the LFO; • D—Referral to community-based initiatives; • E—Intervention at regional initiative: intention for continuation and future activities. • F—Feedback loop in online information platform

At baseline, participants will be asked how they were recruited for the LOFIT study and about their reasons for participation. At 3 months, participants will be asked about their referral from the lifestyle broker and, if applicable, their experiences with the (community-based) initiative they were referred to. At 12 months, intervention participants will be asked about the following: their experiences with the LFO and the care pathway; dose received in terms of contact with the LFO; perceived effect of the program; and support. Reasons for withdrawal or drop-out from the study (measurements) will be registered. After completion of the intervention, participants will be invited for an interview. These interviews will be aimed at understanding the following: reasons for joining the LOFIT study; the impact that the LOFIT study has had on their life; their views on essential program elements for making behavioural change; and their suggestions for improvement of the LFO and care pathway.

Six months after starting the study, all healthcare professionals of the participating departments will receive a short questionnaire about their satisfaction with the LFO. A small sample (≈*n*=2–3 per participating department) will be interviewed to gather opinions and experiences with referral and recruitment to the LOFIT study, and barriers and facilitators for its adoption, implementation and continuation. The research team will join some (research) meetings held by the healthcare professionals and have individual contact with appointed champions to discuss the ongoing process as part of the implementation strategies. Field notes will be taken during these meetings and conversations.

The lifestyle brokers will be asked to complete attendance and online logs. These logs contain information on preparation, delivery and reporting time. Directly after the MI training, they will receive a questionnaire to evaluate the training. At 12 months, all lifestyle brokers will be invited for an in-depth interview to gather their experiences with (delivering) the LFO care, their views on the essential support elements regarding enabling behaviour change, facilitators and barriers for adoption, implementation and continuation of the behaviour change and their suggestions for changes to the novel care model, as well as the respective community-based lifestyle intervention(s). The research team will have regular meetings with lifestyle brokers about work processes. Field notes will be taken during these meetings.

At 12 months, semi-structured interviews will be held with a convenience sample of the community-based lifestyle initiatives to explore perceived barriers and facilitators for implementing the LFO, adherence to protocols and implementation strategies, and overall experiences. For these and all aforementioned interviews, a semi-structured interview guide will be used.

Meetings will occur with stakeholders representing the broader context, such as insurance companies, clinical department managers and general practitioners. Data from these meetings will be recorded in field notes and will be used to evaluate possible facilitators and barriers experienced during the implementation of the LFO.

The extent to which lifestyle brokers delivered the face-to-face session with the patients as intended (i.e. fidelity) will be assessed using the Motivational Interviewing Treatment Integrity (MITI 4.2.1) scale [67]. MITI 4.2.1 is a behavioural coding system that measures the extent to which a practitioner (i.e. lifestyle broker) uses MI skills in a particular session. This instrument is widely used to test MI fidelity and has good reliability and sensitivity [68]. Lifestyle brokers will be asked to audio-record all sessions (after permission of the patient). We aim to code at least four sessions with different patients throughout the randomized controlled trial of each lifestyle broker to provide a reliable competency score on the MITI [69]. To assess an overall MI fidelity score for the LOFIT intervention, we will weigh the individual score of each LSB for the total number of patients counselled.

### Data analysis

#### Effectiveness analyses

Data analyses will be conducted after completion of the data collection at 12 months for both parallel randomized controlled trials separately, no interim analyses are planned. Multilevel linear regression analyses will be performed according the intention-to-treat principle with the adapted Fuster-BEWAT at 12 months as the dependent variable, while study group allocation (i.e. intervention versus usual care) and baseline value of the primary outcome will be modelled as independent variables. Multilevel is chosen to account for clustering of patients on the hospital level. Secondary outcomes will be analysed in a similar way using multilevel linear or logistic regression analyses. Analyses will be performed with STATA SE14. Statistical significance will be set at *p*<0.05.The patterns and extent of missing data will be examined; in case it is necessary, multiple imputations methods will be implemented for primary and secondary outcomes, assuming data are missing at random.

#### Cost-effectiveness analyses

For both parallel randomized controlled trials, cost-effectiveness analyses will be performed separately from both the societal and the healthcare perspective. When the societal perspective is applied, all costs will be included, whereas only those accruing to the formal Dutch healthcare sector will be included when the healthcare perspective is applied. Analyses will be performed according the intention-to-treat principle. In the main analysis, missing cost and effect data will be imputed using Multivariate Imputation by Chained Equations (MICE) [70]. Rubin’s rules will be used to pool the results from the different multiple imputed datasets. Multilevel (i.e. participant level, hospital level) linear regression analyses will be used to estimate cost and effect differences between LOFIT and usual care, while adjusting for confounders if necessary. Incremental cost-effectiveness ratios (ICERs) will be calculated by dividing the differences in costs across groups by the differences in clinical outcomes (i.e. adapted Fuster-BEWAT) and QALYs. Bias-corrected and accelerated bootstrapping with 5000 replications will be used to estimate 95% confidence intervals around the cost differences and statistical uncertainty surrounding the ICERs. Uncertainty surrounding ICERs will be graphically presented on cost-effectiveness planes. Cost-effectiveness acceptability curves will be estimated showing the probability that LOFIT is cost-effective in comparison with usual care for a range of different ceiling ratios, thereby showing decision uncertainty [71]. Sensitivity analyses will be done to assess the robustness of the results.

#### Process evaluation analyses

To evaluate the process data of this study descriptive statistics (mean, SD, proportions) will be used to report patients’, physicians’ and lifestyle brokers’ characteristics and results of pre-structured questions from the questionnaire lifestyle broker logs. All interviews will be audiotaped, fully transcribed verbatim and anonymized. The qualitative data will be analysed using a thematic analysis method [72]. All reported (suggestions and reasons for) adaptations to the program and any other answers to open-ended questions will be listed, analysed and summarized. A framework analysis approach will be used to identify barriers and facilitators for adoption, implementation and continuation, following the TCID-framework of Flottorp et al. [73].

### Oversight and monitoring

The study coordinating centre is Amsterdam UMC. Trial supervision will be conducted by the principal investigator (PI) of the coordinating centre, with weekly meetings across centres. Day-to-day trial management will be supervised by the local PIs, with weekly meetings with local teams. Meetings with champions who integrate the study in daily practice will be scheduled upon request. The Trial Steering Committee is composed of the LOFIT consortium members, which consist of scientific experts, medical doctors and public partners (i.e. health care insurance, applied universities, community-based lifestyle initiatives). Meetings are held every 6 months and used for advice and expertise on topics related to both the practical execution of the RCTs and implementation in the Dutch healthcare system.

#### Protocol amendments

Any protocol amendments that may affect the study design or conduct and patient safety will be submitted to the Amsterdam UMC medical ethical committee for approval. If approved, this will be communicated to the PI of each centre to add to the Investigator Site File. The protocol will be updated in the clinical trial registry. Any protocol deviations will be extensively documented using a deviation log and separate breach report forms for subjects and study deviations.

#### Dissemination

The results of the trial will be submitted to international peer-reviewed journals and presented at national and international conferences. Vancouver convention guidelines for (co) authorship will be applied to all papers by the LOFIT consortium.

## Discussion

The LOFIT study aims to evaluate an in-hospital LFO that provides guidance on lifestyle management in collaboration with community-based lifestyle initiatives for patient under treatment in secondary or tertiary care compared to usual care. At this point in time, there is no such service that is accessible and offers a community-based referral for patients who need it. The novelty of this is thus of great value. This study will show if a lifestyle broker’s additional attention, care and referral to a community-based lifestyle initiative will improve lifestyle behaviour, which, amongst other outcomes, could ultimately cure or reduce disease burden, improve recovery, minimize complications, affect intake of medication and improve quality of life.

Healthcare in the Netherlands, especially care for patients with lifestyle-related NCDs, has a reputation of being fragmented due to the need of different healthcare providers [74, 75]. Integrated care pathways that involve different healthcare professionals and organizations can be successful for patients living with NCDs if coordination and communication are safeguarded [76, 77]. By integrating this innovation in the centre of the process, the lifestyle broker in its role of case manager is not only tailoring care to the individual, but moreover transcends singular professionals, departments and even organizations and their communicational limitations. Communication channels with the lifestyle broker are short which helps to adequately discuss when changes in treatment are necessary as a result of lifestyle changes (e.g. effect on medication intake).

Furthermore, actively involving the patient as part of the team and discussing options with the patient will improve self-management [78]. Previous research has shown that Dutch patients who suffer from NCDs feel that their active involvement is affected by the level of facilitation and empowerment they get, including the understanding of their own health [79]. An intervention that helps people take responsibility for their own health and incorporates being informed in all stages of the journey is a logical element of facilitating patients to be involved actively.

This is not a one-size fits all study where we can evaluate the effectiveness of a single lifestyle intervention. The “cafeteria-like model” of options to choose from in this study will help to make lifestyle medicine more responsive to patient needs and desires. Considerable attention to the context of the patient is provided, whereby not only traditional elements of the healthcare system are included, but also social and municipal services (e.g. debt counselling, addiction treatment) that are associated with health-related factors or a prerequisite for lifestyle change. Such a multidisciplinary and intersectoral approach that transcends different healthcare settings is essential to combating health inequalities [80]. Since a lower social-economic status is related to higher hospital admissions, longer duration of stay and higher costs, there is potential to target patients who are vulnerable for this disparity in health equity in a hospital setting [81].

The information obtained from this pragmatic randomized controlled trial can be used to inform and develop policies regarding attention for and sustainable implementation of lifestyle-related care in secondary and tertiary care. Furthermore, results could provide proof for future health insurance coverage for lifestyle and prevention. Conducting two large, parallel trials targeting highly prevalent NCDs makes a substantial contribution to the current discussion.

## Data Availability

The data supporting the findings of the study will be available upon reasonable request from the corresponding authors (MvD, JJ).

## Abbreviations

AE: Adverse event
ALT: Alanine aminotransferase
ASA: American Society of Anesthesiologists
AST: Aspartate aminotransferase
AUDIT: Alcohol Use Disorders Identification Test
BMI: Body mass index
B-PSQI: Brief Pittsburgh Sleep Quality Index
BRS: Brief Resilience Scale
CCI: Charlson Comorbidity Index
CVD: Cardiovascular disease
DHD15-index: Dutch Healthy Diet index 2015
DM2: Diabetes mellitus type 2
EPD: Electronic Patient Record
FFQ: Food Frequency Questionnaire
FTND: Fagerström Test for Nicotine Dependence
GGT: Gamma-glutamyl transferase
GSE: The General Self-Efficacy scale
HbA1c: Hemoglobin A1C
HDL: High-density lipoprotein cholesterol
HOOS-PS: Hip disability and Osteoarthritis Outcome Score Physical Function Short Form
ICER: Incremental cost-effectiveness ratio
iMCQ: iMTA Medical Consumption Questionnaire
iMTA: Institute for Medical Technology Assessment
IMU: Inertial measurement unit
INT: Interview
IPAQ-SF: International Physical Activity Questionnaire – Short Form
iPCQ: iMTA Productivity Cost Questionnaire
ISI: Insomnia Severity Index
KOOS-PS: Knee injury and Osteoarthritis Outcome Score Physical Function Short Form
LDL: Low-density lipoprotein cholesterol
LFO: Lifestyle front office
LSB: Lifestyle broker
MD: Musculoskeletal disease
MI: Motivational Interviewing
MICE: Multivariate Imputation by Chained Equations
MITI: Motivational Interviewing Treatment Integrity
NCD: Noncommunicable disease
OSA: Obstructive sleep apnea
PI: Principal Investigator
PROM: Patient-related outcome measure
Q: Questionnaire
QALYs: Quality-adjusted life years
RCT: Randomized controlled trial
SBSQ: Set of Brief Screening Questions

## References

[1] Bull FC, Al-Ansari SS, Biddle S, Borodulin K, Buman MP, Cardon G (2020). World Health Organization 2020 guidelines on physical activity and sedentary behaviour. Brit J Sport Med..

[2] World Health Organization. Global status report on noncommunicable diseases 2014. Geneva; 2014. 10.1161/STROKEAHA.115.008097 25873596

[3] World Health Organization (2017). Noncommunicable diseases progress monitor 2017.

[4] Volksgezondheidenzorg.info. Cijfers en achtergronden 2019 [Available from: https://www.volksgezondheidenzorg.info/.

[5] GBD 2016 Disease and Injury Incidence and Prevalence Collaborators. Global, regional, and national incidence, prevalence, and years lived with disability for 328 diseases and injuries for 195 countries, 1990-2016: a systematic analysis for the Global Burden of Disease Study 2016. Lancet, 59. 2017;390(10100): 1211 10.1016/S0140-6736(17)32154-2 PMC5605509 28919117

[6] World Health Organization. Obesity and Overweight 2018 http://www.who.int/mediacentre/factsheets/fs311/en/.

[7] Pampel FC, Krueger PM, Denney JT (2010). Socioeconomic disparities in health behaviors. Annu Rev Sociol.

[8] Bassi N, Karagodin I, Wang S, Vassallo P, Priyanath A, Massaro E (2014). Lifestyle modification for metabolic syndrome: a systematic review. Am J Med..

[9] Astin F, Horrocks J, Closs SJ (2014). Managing lifestyle change to reduce coronary risk: a synthesis of qualitative research on peoples' experiences. Bmc Cardiovasc Disor..

[10] Nauta J, van Nassau F, Bouma AJ, Krops LA, van der Ploeg HP, Verhagen E (2022). Facilitators and barriers for the implementation of exercise are medicine in routine clinical care in Dutch university medical centres: a mixed methodology study on clinicians' perceptions. BMJ Open.

[11] Harkin N, Johnston E, Mathews T, Guo Y, Schwartzbard A, Berger J (2019). Physicians' dietary knowledge, attitudes, and counseling practices: the experience of a single health care center at changing the landscape for dietary education. Am J Lifestyle Med..

[12] Krops LA, Bouma AJ, Van Nassau F, Nauta J, van den Akker-Scheek I, Bossers WJ (2020). Implementing individually tailored prescription of physical activity in routine clinical care: protocol of the Physicians Implement Exercise = Medicine (PIE=M) Development and Implementation Project. JMIR Res Protoc..

[13] Smith AW, Borowski LA, Liu BM, Galuska DA, Signore C, Klabunde C (2011). US primary care physicians' diet-, physical activity-, and weight-related care of adult patients. Am J Prev Med..

[14] Van de Glind IM, Heinen MM, Geense WW, Mesters I, Wensing M, Van Achterberg T (2016). Exploring the range of lifestyle interventions used in Dutch health care practice: a qualitative description. Health Promot Pract..

[15] VanBuskirk KA, Wetherell JL (2014). Motivational interviewing with primary care populations: a systematic review and meta-analysis. J Behav Med..

[16] Coffeng JK, van der Ploeg HP, Castellano JM, Fernandez-Alvira JM, Ibanez B, Garcia-Lunar I (2017). A 30-month worksite-based lifestyle program to promote cardiovascular health in middle-aged bank employees: Design of the TANSNIP-PESA randomized controlled trial. Am Heart J..

[17] Garcia-Lunar I, van der Ploeg HP, Fernandez Alvira JM, van Nassau F, Castellano Vazquez JM, van der Beek AJ (2022). Effects of a comprehensive lifestyle intervention on cardiovascular health: the TANSNIP-PESA trial. Eur Heart J..

[18] Sealed Envelope Ltd. Sealed Envelope - create a blocked randomisation list 2021 [Available from: https://www.sealedenvelope.com/simple-randomiser/v1/lists.

[19] Van der Pluijm S. Coachen 3.0 - deel 1. Motiverende gespreksvoering 4th edition ed. www.hetboekenschap.nl: Het Boekenschap; 2021.

[20] Michie S, van Stralen MM, West R (2011). The behaviour change wheel: a new method for characterising and designing behaviour change interventions. Implement Sci..

[21] Miller WR, Rollnick S (2012). Motivational interviewing - helping people change.

[22] Miller WR, Rollnick S (2014). The effectiveness and ineffectiveness of complex behavioral interventions: Impact of treatment fidelity. Contemp Clin Trials.

[23] Miller WRR, S. (1991). Motivational interviewing: preparing people to change addictive behavior.

[24] Fernandez-Alvira JM, Fuster V, Pocock S, Sanz J, Fernandez-Friera L, Laclaustra M (2017). Predicting subclinical atherosclerosis in low-risk individuals: ideal cardiovascular health score and Fuster-BEWAT score. J Am Coll Cardiol..

[25] Saunders JB, Aasland OG, Babor TF, de la Fuente JR, Grant M (1993). Development of the Alcohol Use Disorders Identification Test (AUDIT): WHO Collaborative Project on Early Detection of Persons with Harmful Alcohol Consumption--II. Addiction.

[26] Schippers GM, Broekman TG. De AUDIT. Nederlandse vertaling van de Alcohol Use Disorders Identification Test. 2010.

[27] Marshall AL, Miller YD, Burton NW, Brown WJ (2010). Measuring total and domain-specific sitting: a study of reliability and validity. Med Sci Sports Exerc..

[28] Blikman T, Stevens M, Bulstra SK, van den Akker-Scheek I, Reininga IH (2013). Reliability and validity of the Dutch version of the International Physical Activity Questionnaire in patients after total hip arthroplasty or total knee arthroplasty. J Orthop Sports Phys Ther..

[29] Craig CL, Marshall AL, Sjostrom M, Bauman AE, Booth ML, Ainsworth BE (2003). International physical activity questionnaire: 12-country reliability and validity. Med Sci Sports Exerc..

[30] Lee PH, Macfarlane DJ, Lam TH, Stewart SM (2011). Validity of the International Physical Activity Questionnaire Short Form (IPAQ-SF): a systematic review. Int J Behav Nutr Phys Act..

[31] Meijer RvH N, Papen-Botterhuis NE, Molenaar CJL, Regis M, Timmers T, Van de Poll-Franse LV, et al. Estimating VO2peak in 18-91 year-old adults | Development and validation of the FitMáx© - Questionnaire (Preprint). ACSM: Med & Sci in Sports & Ex. 2021. 10.2147/IJGM.S355589 PMC8994663 35411174

[32] Bastien CH, Vallieres A, Morin CM (2001). Validation of the Insomnia Severity Index as an outcome measure for insomnia research. Sleep Med..

[33] Morin CM, Belleville G, Belanger L, Ivers H (2011). The Insomnia Severity Index: psychometric indicators to detect insomnia cases and evaluate treatment response. Sleep..

[34] Thorndike FP, Ritterband LM, Saylor DK, Magee JC, Gonder-Frederick LA, Morin CM (2011). Validation of the insomnia severity index as a web-based measure. Behav Sleep Med..

[35] Sancho-Domingo C, Carballo JL, Coloma-Carmona A, Buysse DJ (2021). Brief version of the Pittsburgh Sleep Quality Index (B-PSQI) and measurement invariance across gender and age in a population-based sample. Psychol Assess..

[36] Chung F, Subramanyam R, Liao P, Sasaki E, Shapiro C, Sun Y (2012). High STOP-Bang score indicates a high probability of obstructive sleep apnoea. Br J Anaesth..

[37] Chung F, Yegneswaran B, Liao P, Chung SA, Vairavanathan S, Islam S (2008). STOP questionnaire: a tool to screen patients for obstructive sleep apnea. Anesthesiology..

[38] Heatherton TF, Kozlowski LT, Frecker RC, Fagerstrom KO (1991). The Fagerstrom Test for Nicotine Dependence: a revision of the Fagerstrom Tolerance Questionnaire. Br J Addict..

[39] Cantril H (1966). Pattern of human concerns.

[40] Herdman M, Gudex C, Lloyd A, Janssen MF, Kind P, Parkin D (2011). Development and preliminary testing of the new five-level version of EQ-5D (EQ-5D-5L). Qual Life Res..

[41] Versteegh MM, Vermeulen KM, Evers SM, de Wit GA, Prenger R, Stolk EA (2016). Dutch Tariff for the Five-Level Version of EQ-5D. Val Health..

[42] Smith BW, Dalen J, Wiggins K, Tooley E, Christopher P, Bernard J (2008). The brief resilience scale: assessing the ability to bounce back. Int J Behav Med..

[43] Jerusalem M, Schwarzer R (1992). Self-efficacy as a resource factor in stress appraisal processes, self-efficacy: thought control and action.

[44] Teeuw B, Schwarzer R, Jerusalem M (1994). Dutch Adaptation of the General Self-Efficacy Scale.

[45] Prochaska JO, Redding CA, Evers KE, Rimer BK, Viswanath K, Glanz K (2008). The transtheoretical model and stage of change. Health Behavior and Health Education Theory, Research and Practice.

[46] de Groot IB, Favejee MM, Reijman M, Verhaar JA, Terwee CB (2008). The Dutch version of the Knee Injury and Osteoarthritis Outcome Score: a validation study. Health Qual Life Out.

[47] Chew LD, Bradley KA, Boyko EJ (2004). Brief questions to identify patients with inadequate health literacy. Fam Med..

[48] American Society of Anesthesiologists. ASA Phy Stat Classifi 2020:https://www.asahq.org/standards-and-guidelines/asa-physical-status-classification-system.

[49] Charlson ME, Carrozzino D, Guidi J, Patierno C (2022). Charlson Comorbidity Index: a critical review of clinimetric properties. Psycho Psychos..

[50] Bouwmans C, Krol M, Severens H, Koopmanschap M, Brouwer W, Hakkaart-van RL (2015). The iMTA Productivity Cost Questionnaire: a standardized instrument for measuring and valuing health-related productivity losses. Val Health.

[51] Moore GF, Audrey S, Barker M, Bond L, Bonell C, Hardeman W (2015). Process evaluation of complex interventions: Medical Research Council guidance. BMJ..

[52] Alberti KG, Eckel RH, Grundy SM, Zimmet PZ, Cleeman JI, Donato KA (2009). Harmonizing the metabolic syndrome: a joint interim statement of the International Diabetes Federation Task Force on Epidemiology and Prevention; National Heart, Lung, and Blood Institute; American Heart Association; World Heart Federation; International Atherosclerosis Society; and International Association for the Study of Obesity. Circulation.

[53] Nederlands Huisartsen Genootschap. NHG-standaard: Laboratoriumdiagnostiek Leveraandoeningen (LESA) 2019 [Available from: https://www.nhg.org/themas/publicaties/laboratoriumdiagnostiek-leveraandoeningen-volledige-tekst.

[54] Nederlands Huisartsen Genootschap. NHG-standaard: Chronische nierschade 2018 [Available from: https://richtlijnen.nhg.org/standaarden/chronische-nierschade.

[55] Nederlands Huisartsen Genootschap. NHG-standaard: Diabetes mellitus type 2 2018 [Available from: https://richtlijnen.nhg.org/standaarden/diabetes-mellitus-type-2.

[56] Nederlands Huisartsen Genootschap. NHG-standaard: Cardiovasculair risicomanagement 2019 [Available from: https://richtlijnen.nhg.org/standaarden/cardiovasculair-risicomanagement.

[57] Ashwell M, Gunn P, Gibson S (2012). Waist-to-height ratio is a better screening tool than waist circumference and BMI for adult cardiometabolic risk factors: systematic review and meta-analysis. Obes Rev..

[58] Ben-Noun L, Sohar E, Laor A (2001). Neck circumference as a simple screening measure for identifying overweight and obese patients. Obes Res..

[59] Swartz AM, Rote AE, Cho YI, Welch WA, Strath SJ (2014). Responsiveness of motion sensors to detect change in sedentary and physical activity behaviour. Br J Sports Med..

[60] Eussen SJ, van Dongen MC, Wijckmans NE, Meijboom S, Brants HA, de Vries JH (2018). A national FFQ for the Netherlands (the FFQ-NL1.0): development and compatibility with existing Dutch FFQs. Public Health Nutr..

[61] Looman M, Feskens EJ, de Rijk M, Meijboom S, Biesbroek S, Temme EH (2017). Development and evaluation of the Dutch Healthy Diet index 2015. Pub Health Nutr..

[62] Papadaki A, Johnson L, Toumpakari Z, England C, Rai M, Toms S (2018). Validation of the English Version of the 14-Item Mediterranean Diet Adherence Screener of the PREDIMED Study, in People at High Cardiovascular Risk in the UK. Nutrients.

[63] Gezondheidsraad. (2015). Richtlijnen goede voeding 2015.

[64] Dahlberg LE, Team IHKO (2016). Ichom Standard Set for Monitoring Knee and Hip Osteoarthritis. Osteo Cartil.

[65] Gossec L, Paternotte S, Maillefert JF, Combescure C, Conaghan PG, Davis AM (2011). The role of pain and functional impairment in the decision to recommend total joint replacement in hip and knee osteoarthritis: an international cross-sectional study of 1909 patients. Report of the OARSI-OMERACT Task Force on total joint replacement. Osteo Cartil.

[66] Hakkaart-van Roijen L, Van der Linden N, Bouwmans C, Kanters T, Tan SS. Kostenhandleiding. Methodologie van kostenonderzoek en referentieprijzen voor economische evaluaties in de gezondheidszorg In opdracht van Zorginstituut Nederland Geactualiseerde versie. 2015:12-64. .

[67] Moyers TB, Manuel JK, Ernst D. Motivational Interviewing Treatment Integrity Coding Manual 4.2.1 (MITI 4.2.1). Unpublished manual; 2014. Microsoft Word - MITI 4.2.1 June 2015.docx (unm.edu).

[68] Forsberg L, Berman AH, Kallmen H, Hermansson U, Helgason AR (2008). A test of the validity of the motivational interviewing treatment integrity code. Cogn Behav Ther..

[69] Jelsma JG, Mertens VC, Forsberg L, Forsberg L (2015). How to measure motivational interviewing fidelity in randomized controlled trials: practical recommendations. Contemp Clin Trials.

[70] Van Buuren S, Boshuizen HC, Knook DL (1999). Multiple imputation of missing blood pressure covariates in survival analysis. Stat Med..

[71] Fenwick E, O'Brien BJ, Briggs A (2004). Cost-effectiveness acceptability curves - facts, fallacies and frequently asked questions. Health Econ..

[72] Castleberry A, Nolen A (2018). Thematic analysis of qualitative research data: is it as easy as it sounds?. Curr Pharm Teach Learn..

[73] Flottorp SA, Oxman AD, Krause J, Musila NR, Wensing M, Godycki-Cwirko M, et al. A checklist for identifying determinants of practice: a systematic review and synthesis of frameworks and taxonomies of factors that prevent or enable improvements in healthcare professional practice. Implement Sci. 2013;8. (1), 1-1 10.1186/1748-5908-8-35 PMC3617095 23522377

[74] Kroneman M, Boerma W, van den Berg M, Groenewegen P, de Jong J, van Ginneken E (2016). Health systems in transition. Health..

[75] Nolte E, Knai C, Hofmarcher M, Conklin A, Erler A, Elissen A, Flamm M, Fullerton B, Sönnichsen A, Vrijhoef HJ (2012). Overcoming fragmentation in health care: chronic care in Austria, Germany and The Netherlands. Health Eco, Pol and Law.

[76] Provost S, Pineault R, Grimard D, Perez J, Fournier M, Levesque Y (2017). Implementation of an integrated primary care cardiometabolic risk prevention and management network in Montreal: does greater coordination of care with primary care physicians have an impact on health outcomes?. Health Promot Chronic Dis Prev Can..

[77] Lee JK, McCutcheon LRM, Fazel MT, Cooley JH, Slack MK (2021). Assessment of Interprofessional Collaborative Practices and Outcomes in Adults With Diabetes and Hypertension in Primary Care: a systematic review and meta-analysis. JAMA Netw Open..

[78] Holman H, Lorig K (2000). Patients as partners in managing chronic disease - partnership is a prerequisite for effective and efficient health care. Brit Med J..

[79] Buljac-Samardzic M, Clark MA, van Exel NJA, van Wijngaarden JDH (2022). Patients as team members: factors affecting involvement in treatment decisions from the perspective of patients with a chronic condition. Health Exp.

[80] Garzon-Orjuela N, Samaca-Samaca DF, Luque Angulo SC, Mendes Abdala CV, Reveiz L, Eslava-Schmalbach J (2020). An overview of reviews on strategies to reduce health inequalities. Int J Equity Health..

[81] Yong J, Yang O (2021). Does socioeconomic status affect hospital utilization and health outcomes of chronic disease patients?. Eur J Health Econ..

